# Shear-Induced and Nanofiber-Nucleated Crystallization of Novel Aliphatic–Aromatic Copolyesters Delineated for In Situ Generation of Biodegradable Nanocomposites

**DOI:** 10.3390/polym13142315

**Published:** 2021-07-14

**Authors:** Ramin Hosseinnezhad

**Affiliations:** Centre of Molecular and Macromolecular Studies, Polish Academy of Sciences, 90-363 Lodz, Poland; ramin.h@cbmm.lodz.pl

**Keywords:** shear-induced crystallization, copolyesters, nanocomposite, crystallization temperature, lamellae

## Abstract

The shear-induced and cellulose-nanofiber nucleated crystallization of two novel aliphatic–aromatic copolyesters is outlined due to its significance for the in situ generation of biodegradable nanocomposites, which require the crystallization of nanofibrous sheared inclusions at higher temperatures. The shear-induced non-isothermal crystallization of two copolyesters, namely, poly(butylene adipate-co-succinate-co-glutarate-co-terephthalate) (PBASGT) and poly(butylene adipate-co-terephthalate) (PBAT), was studied following a light depolarization technique. To have a deep insight into the process, the effects of the shear rate, shear time, shearing temperature and cooling rate on the initiation, kinetics, growth and termination of crystals were investigated. Films of 60 μm were subjected to various shear rates (100–800 s^−1^) for different time intervals during cooling. The effects of the shearing time and increasing the shear rate were found to be an elevated crystallization temperature, increased nucleation density, reduced growth size of lamella stacks and decreased crystallization time. Due to the boosted nucleation sites, the nuclei impinged with each other quickly and growth was hindered. The effect of the cooling rate was more significant at lower shear rates. Shearing the samples at lower temperatures, but still above the nominal melting point, further shifted the non-isothermal crystallization to higher temperatures. As a result of cellulose nanofibers’ presence, the crystallization of PBAT, analyzed by DSC, was shifted to higher temperatures.

## 1. Introduction

Due to recent increased awareness about the global environment, a new concept of in situ generation of all-polymeric biodegradable nanocomposites has been developed, where the nanofibers of one biodegradable polymer are formed inside a second biodegradable polymer during melt compounding. The solidification of nanofibrous sheared inclusions is achieved by shear-induced crystallization during processing. The concept has been applied to a range of biodegradable polymers, namely, polylactide (PLA), polyhydroxyalkanoate (PHA), poly(glycolic acid) (PGA), poly(butylene adipate-co-terephthalate) (PBAT), bio-based polyamide (PA), poly(1,4-butylene succinate) (PBS) and poly(ε-caprolactone) (PCL), to fabricate sustainable, green, all–polymer nanocomposites in a single stage. The critical role of applying a high shear rate to precisely control the phase morphology of the dispersed polymer inclusions has been revealed [1,2,3,4,5,6,7,8].

Typically, such crystallization of biodegradable polymers during practical melt compounding is realized under non-isothermal conditions, more preferably at high cooling rates. However, practical attempts to obtain induced chain orientation and enhanced crystallization with point-like or fibrillar nuclei could be challenging due to the remarkable impacts of the shear temperature, shear rate and total strain on the final structure [9,10,11]. High molecular weight (HMW) polymers with longer chains are more vulnerable to shearing, showing more efficiently induced crystallization. The structure of induced nuclei, point-like or fibrillar, was augmented via applying higher shear rates to HMW polymers. The obtained improved structures were also tentatively attributed to processing mechanical conditions [12,13].

Recently, aliphatic–aromatic copolyesters (AACs) among biodegradable polymers have been accurately designated for toughening PLA-based biodegradable nanocomposites since the balance between biodegradability and desirable physical properties of AACs can be tailored by controlling the molar ratio of comonomers [14,15,16,17]. Poly (butylene adipate-co-terephthalate) (PBAT), a highly flexible biodegradable aliphatic–aromatic copolyester, has been blended with PLA to improve the flexibility and processability of PLA for biodegradable functional packaging applications [18,19,20]. Notwithstanding the urge to understand the crystallization kinetics of AACs for the in situ generation of biodegradable nanocomposites, the shear-induced crystallization of these copolyesters has not been investigated well. A study investigating the crystallization of a newly synthesized aliphatic–aromatic copolyester (PBAGST) based on three aliphatic comonomers (succinate, glutarate and adipate dibutyl esters) and one aromatic (butylene terephthalate) comonomer revealed that the aromatic self-nucleation sites are stable below 150 °C, which is far lower than industrial compounding conditions. As soon as PBASGT is melted at a higher temperature, these self-nucleation sites disappear and the subsequent crystallization temperature drops by 25 °C to lower temperatures [21,22]. The latest ex situ crystallization study of PBAT using polarized light microscopy suggests a great possibility for shifting crystallization during cooling to higher temperatures, with an enhanced point-like nucleated structure [23].

Studying the effect of cellulose nanofibers (CNFs) on the non-isothermal crystallization behavior of PBAT is also of great importance for the in situ generation of hybrid all-polymeric biodegradable nanocomposites, where the crystallization temperature of PBAT nanofibrous sheared inclusions could be further shifted to higher temperatures due to the presence of cellulose bio-nanofibers. This idea stems from the recent studies reporting that CNFs could act as crystal nucleating agents, increasing the number of crystals and decreasing the crystal sizes for the non-isothermal crystallization of biodegradable polymers [24].

To the best of our knowledge, the effect of CNFs on the crystallization of PBAT for the in situ generation of hybrid all-biopolymeric nanocomposites had not been considered. Furthermore, a clear understanding of the non-isothermal crystallization behavior of AACs is indispensable since the in situ nano-fibrillation of AACs within another biodegradable polymer is achieved under non-isothermal conditions during compounding. Hence, it is crucial to establish the relationship between processing and the induced crystallization at higher temperatures. In particular, the significance of this study is to examine the effect of various processing conditions and the presence of CNFs on the non-isothermal crystallization kinetics of AACs for the in situ shear-induced production of hybrid all-polymeric nanocomposites.

In the present paper, the great importance of shear-induced crystallization for the in situ generation of nanocomposites is explored. To have a deep insight into the process, we investigated the effects of the shear rate, shear time, shearing temperature and cooling rate on the initiation, kinetics, growth and termination of crystals. The shear-induced non-isothermal crystallization of two copolyesters, PBAT and PBASGT, was scrutinized in situ using polarized light microscopy (PLM) and ex situ, employing differential scanning calorimetry (DSC). Finally, the crystallization of PBAT in the presence of cellulose nanofibers as an induced nucleating agent were studied with DSC.

## 2. Experimental

### 2.1. Materials

Poly(butylene adipate-co-succinate-co-glutarate-co-terephthalate) (PBASGT), a synthesized aliphatic–butylene terephthalate copolyester, was used upon the synthesis of a mixture of aliphatic dicarboxylic acid dimethyl esters, which contained 31% dimethyl adipate (DMA), 14% dimethyl succinate (DMS), 55% dimethyl glutarate (DMG) and dimethyl terephthalate in the proportion of 60% concerning the mixture of aliphatic components. This aliphatic–aromatic copolyester was prepared by a two-step melt polycondensation method with Ti(OBu)_4_ as the catalyst. The details of the reaction are described as follows: into a 30 dm^3^ acid-proof clave warmed to 140 °C with a nitrogen atmosphere, 3500 g of DMT, 5600 g of BD, 4400 g of Uniestrol and 2.5 g of Ti(OBu)_4_ as a catalyst were introduced. The clave was then warmed up to a temperature in the range of 165 °C to 225 °C under atmospheric pressure. Simultaneously, methanol was distilled out of the clave. The transesterification was completed within about 100 min. Subsequently, 3.5 g of Ti(OBu)_4_ was added, the pressure was slowly decreased to 0.53 hPa, and the reaction temperature was raised to 250 °C. An excess of 1,4-butanediol was distilled in this period. The total time for the polycondensation process was 120 min. The molten polymer was extruded in the form of 2–3 mm thick strings and pelletized [21]. The final product consisted of 62.7% of the aromatic component as was determined from the ^1^H NMR spectrum. The distribution of aromatic units along the macromolecular chains was estimated from ^1^H NMR and was nearly random, as judged from the randomness index of 1.005, where 1 is characteristic for complete randomness [21]. The molecular weights as measured by SEC-MALLS were Mw = 53,700, Mn = 27,700 and Mz = 189,000. Poly(butylene adipate-co-terephthalate) (PBAT) with the trade name Ecoflex type F Blend C1200 was purchased from BASF SE (Ludwigshafen am Rhein, Germany). Cellulose nanofibers (CNFs) with diameters between 10 and 20 nm and lengths of 2 to 3 µm, obtained from Nanografi (Ankara, Turkey), were employed to enhance the crystallization of PBAT.

### 2.2. Sample Preparation

PBAT and PBASGT films with an approximate thickness of 100 μm were prepared using a hot press, where the samples were compressed at 180 °C for 5 min and subsequently cooled down to ambient temperature. The final thickness of the hot-pressed films was measured by using a micrometer (Sylvac SA, Yverdon, Switzerland), with a resolution of 1 µm. Melt blends containing 5, 10 and 20 wt.% of CNFs and PBAT were homogenized using a Brabender mixer (Duisburg, Germany) at 60 rpm for 5 min at 180 °C. To avoid degradation due to hydrolysis, PBAT was dried under vacuum at 60 °C for 8 h before blending with cellulose nanofibers.

### 2.3. Rheo-Optical Measurements: Shear-Induced Crystallization Test

**Figure 1:**
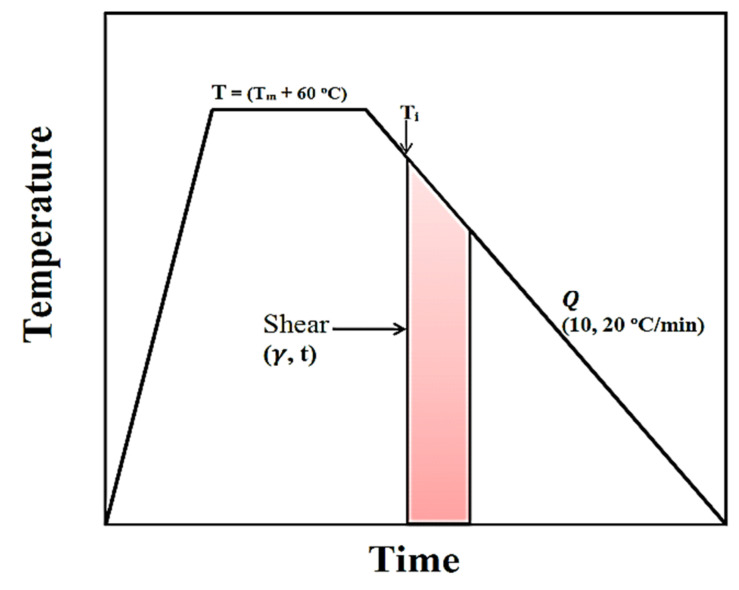
Schematic indication of thermal and shear treatment for crystallization process.

A Linkam optical shearing system (CSS450, Waterfield, UK) was used to control the shear and thermal programs applied to the films. It was mounted in a polarizing light microscope Eclipse 80i (Nikon, Buckinghamshire, UK) equipped with a video camera DS-Fil (Nikon, Buckinghamshire, UK) for monitoring crystallization. Figure 1 represents the general schematic of thermal and shear application for the crystallization process. The films were heated to 60 °C above the melting point at 30 °C/min, held for 5 min to erase the thermal history while the caliper was set to 60 μm and subsequently cooled down at rates of 10 and 20 °C/min. To ensure a uniform thickness of the samples during shearing, the gap in the shearing cell between the upper and lower sections was set to 60 µm and controlled by a stepper motor and gap mechanism during shearing. During cooling, the samples were subjected to various shear rates (100–800 s^−1^) for different durations (30, 45, 60 and 90 s). The shearing program was initiated at different temperatures. The shearing conditions were selected based on preliminary studies. Control specimens, with the same thermal history as the sheared ones, were crystallized in quiescent conditions. In each case, at least three specimens were crystallized and examined. The development of structures during crystallization was monitored with PLM.

The conversion of melt into the crystalline phase was followed by the light depolarization method. The volume conversion degree at temperature T, α(T), was calculated according to the following equation:

α (T) = [I(T) − I(T_0_)]/[I(T_e_) − I(T_0_)] (1)

where I(T) is the intensity of the transmitted depolarized light at temperature T, whereas T_0_ and Te denote the initial temperature and final temperature to which the sample was cooled.

### 2.4. Thermal Analysis

The thermal behavior of the blends was probed with a DSC Q20 (TA Instruments, New Castle, DE, USA) during heating from −100 °C to 220 °C at a heating rate of 10 °C/min, isotherm at 220 °C for 3 min and subsequent cooling down to −50 °C at the rate of 10 °C/min. Samples of 7–8 mg in mass were cut out from PBASGT/CNF blends and crimped in standard Al pans. The DSC cell was purged with dry nitrogen during the measurements (20 mL/min).

Thermal gravimetric analysis (TGA) experiments were conducted with a TA Instruments Hi-Res TGA 2950 Thermogravimetric Analyzer (New Castle, DE, USA) at the heating rate of 10 °C/min. It was used to thermally decompose samples under controlled heating and environmental conditions in nitrogen to detect their thermal stability and weight reduction. Two types of plots were produced. A plot of specimen weight against temperature (TGA curve) provided the thermal decomposition temperatures, with the residue amount as a function of temperature. The second plot (DTG), a derivative of the TGA curve, indicated a mass loss rate dependent on an increase in temperature.

## 3. Results

### 3.1. Shear-Induced Crystallization under Various Process Conditions

The crystallization kinetics of AACs, as a function of the shear rate and crystallization temperature, could also be investigated during isotherm experiments by using an equation including shearing and relaxation effects. Due to more accurate temperature control, the study may have resulted in a comprehensive prediction of crystallization behavior. However, the relaxation effect may have predominated over the influence of shear during the isotherm crystallization.

The essence of this work is based on comparative results for revealing the effects of different parameters (e.g., the shear rate, shearing temperature, shearing time, content of CNF, etc.) on the shear-induced non-isothermal crystallization of AACs. The conclusions are drawn based on comparing the crystallization curves (e.g., degree of conversion versus temperature) collected under the same cooling condition. Even though the reported crystallization rates (characterized by T_c_) might differ from the real values due to the inherent challenges of temperature control, the trend of variation is valid for drawing conclusions. The present study did not attempt to analyze the experimental data using theoretical models, nor did it attempt to verify any theory of polymer crystallization. For the sake of identifying a general trend or effect of the empirical parameters in a qualitative or semi-quantitative manner, the experiments suffice for the purpose.

#### 3.1.1. Effect of Shear Rate on T_c_

**Figure 2:**
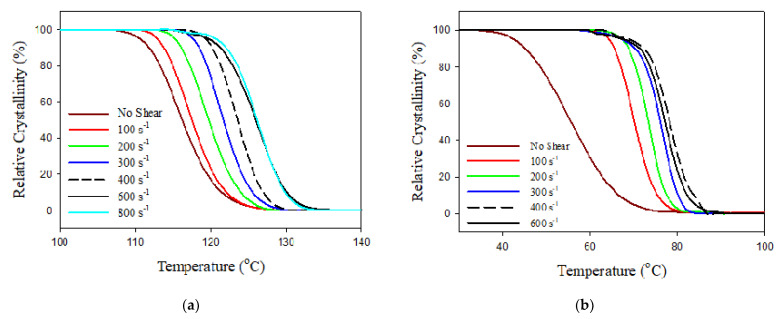
Temperature dependencies of conversion degree for (**a**) PBASGT and (**b**) PBAT specimens sheared for 45 s during cooling at 20 °C/min. The shearing with different rates in the range of 100–800 s^−1^ was initiated at 220 °C.

Figure 2 exhibits the remarkable temperature reliance of the conversion degree measured during the non-isothermal crystallization of PBAT and PBASGT induced at higher temperatures by applying different shear rates. As shown in the figure, the shear deformation of molten samples prior to crystallization, in both PBAT and PBASGT, generally elevates the temperature at which transition to solid-state occurs. The effect of the shear rate on the crystallization temperature, T_c_, of films sheared for 45 s is remarkable. It is revealed that, upon shearing with 100 s^−1^, the transition initiates, develops and terminates at higher temperatures, resulting in uplifted T_c_.

The crystallization temperature, meaning the temperature at which the conversion degree reaches 50%, is 116 °C for non-sheared PBASGT. Increasing the shearing rate leads to a gradual shift in the crystallization temperature of PBASGT towards higher temperatures and a reduction in the temperature range, ΔT, in which the transition from a molten state to a crystallized one occurs. The maximum effect is achieved at 800 s^−1^.

#### 3.1.2. Effect of Shear Rate on Crystal Growth

**Figure 3:**
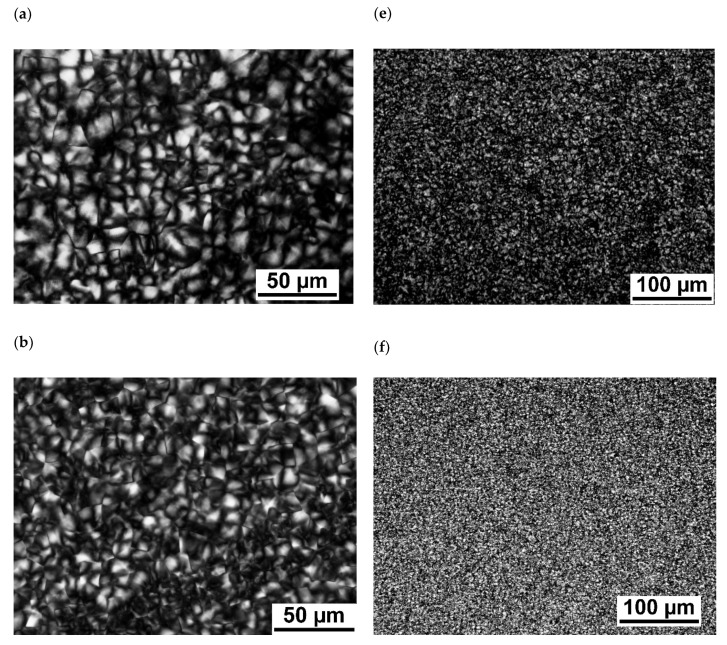

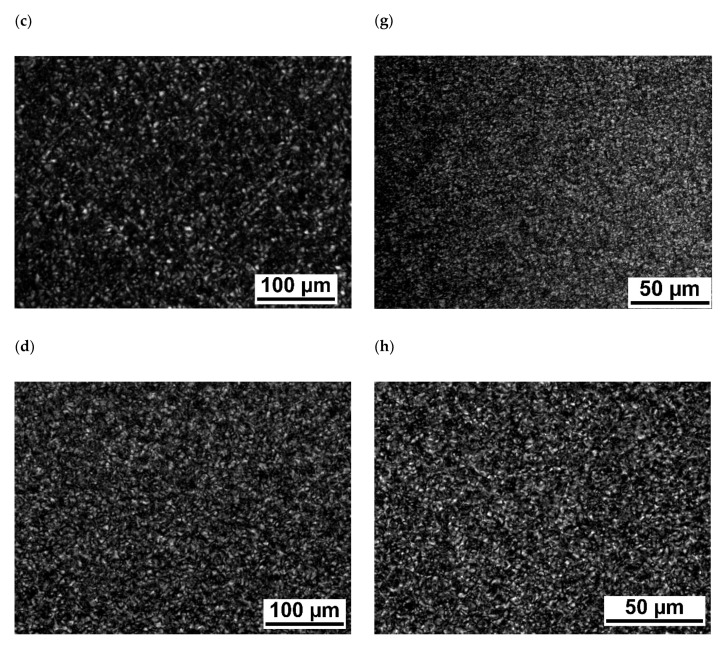
PLM micrographs of PBASGT specimens: not sheared (**a**) and sheared for 45 s at 100 s^−1^ (**b**), 200 s^−1^ (**c**), and 300 s^−1^ (**d**); PBAT specimens: not sheared (**e**) and sheared for 45 s at 100 s^−1^ (**f**), 200 s^−1^ (**g**), and 300 s^−1^ (**h**).

Figure 3 depicts PLM micrographs of PBAT and PBASGT specimens not sheared and sheared for 45 s at 100, 200 and 300 s^−1^. The PLM micrograph of the non-sheared PBASGT specimen, Figure 3a, exhibits the spherulite morphology of nucleated PBASGT formed during non-isothermal crystallization. The spherulites were well-grown, filling the entire volume of the film. The average size of the spherulites was approximately 13 µm. Shearing the samples altered the density of nucleated spherulites as well as the growth. The sizes of the nucleated spherulites were comparatively smaller for the sheared samples. Additionally, the nucleation density for the sheared film was much higher than that for the non-sheared sample. The PLM micrographs in Figure 3b–d affirm the increase in nucleation density and the decrease in spherulite size upon increasing the shear rate from 100 to 300 s^−1^. Increasing the employed shear rate decreased the growth of many small spherulites. Sheared samples were filled with these fine spherulites. The size of spherulites in the samples sheared with 300 s^−1^ was decreased to 5 µm, as resolved in polarized light micrographs. However, even applying the high shear rate of 300 s^−1^ to the PBASGT melted sample did not affect its one-directional irregular growth of lamellar spherulites. Figure 3e–h reveals the similar effect of γ˙ on the size and density of polycrystalline aggregates of PBAT specimens. It is seen that polycrystalline aggregates of PBAT had smaller sizes compared to PBASGT. In addition, the effect of shear on increasing the number of nuclei for PBAT is less remarkable. The average size of aggregates for the non-sheared PBAT is approximately 7 µm and decreases to 3–4 µm for the sample sheared with 300 s^−1^. It is suggested that shear stress affects the nucleation rate of PBAT rather than the growth of its crystals. In any case, the final size of spherulites in the sheared samples was smaller.

#### 3.1.3. Effect of Shear Rate on Crystallization Kinetics

**Figure 4:**
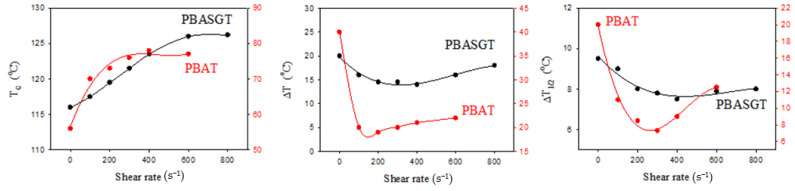
Shear rate dependences of T_c_, ΔT and ΔT_1/2_ of PBAT and PBASGT, crystallized from the melt. The samples were cooled down with simultaneous shearing at various shearing rates.

Figure 4 shows the shear rate dependencies of the crystallization temperature T_c_, ΔT_1/2_ and ΔT for PBAT and PBASGT. The term ΔT represents the temperature range over which full transition from the molten to crystalline state occurs, while ΔT_1/2_ refers to the temperature where there is 50% conversion. The crystalline structure of non-sheared PBAT films forms slowly (40 s), in comparison to the crystal growth of PBASGT (20 s). It is found that, once the specimens are sheared during cooling, the conversion of molten samples to crystals could initiate and grow over a higher and narrower range of temperatures. This demonstrates remarkable shifts in the crystallization temperature of PBAT and PBASGT, induced via the applied shear rates. As demonstrated in Figure 4a, shearing PBAT films for 45 s with a shear rate of 100 s^−1^ resulted in a significant increase in T_c_ from 56 °C for the non-sheared sample to 70 °C. Moreover, ΔT decreased from 42 °C to 20 °C. The crystallization temperature was raised to 78 °C upon applying shear with the rate 400 s^−1^. However, the insufficient melt flow strength of PBAT and non-laminar flow due to Taylor vortices challenged the realization of crystallization kinetics at a shear rate above 400 s^−1^. Similarly, the inception of crystallization for PBASGT shifted from 116 °C to 126 °C. Meanwhile, the crystallization was accomplished (ΔT) over a shorter range of 25 °C rather than 40 °C for the non-sheared one, as shown in Figure 4b. In the case of PBASGT, T_c_ was shifted approximately 2 °C to higher temperatures for each increment of 100 s^−1^ in shear rate. This is due to the fact that molten chains may orient easily to crystallize and the growth quickly stabilizes at a smaller size. No such decrease in the time required for crystallization to proceed was observed for sheared PBASGT samples. Despite sheared PBAT crystallizing within a narrower temperature range, ΔT remained almost constant for PBASGT while ΔT_1/2_ decreased. It seems that the orientational memory of chains is preserved over a small time gap between the cessation of shearing and the onset of crystallization leading to the decrease in ΔT_1/2_. Once 50% conversion is accomplished, the loss of conformation memory retards the growth of nuclei. The results indicate that the temperature for the first spherulite nucleation shifts from 80 °C to 90 °C for PBAT and shifts from 125 °C to 136 °C for PBASGT. In comparison to the 21 °C shift in T_c_ towards higher temperatures for PBAT, a smaller shift of 10 °C was observed for PBASGT sheared at the rate of 600 s^−1^ for 45 s.

#### 3.1.4. Effect of Cooling Rate on T_c_

Table 1 summarizes the T_c_ values obtained for PBAT and PBASGT specimens after being sheared for 45 s at 10 and 20 °C/min. The shifts in the crystallization temperature are smaller for the higher cooling rate. As the cooling rate decreases, both PBAT and PBASGT samples nucleate and initiate crystallization at higher temperatures. Additionally, the crystal size increased slightly upon decreasing the cooling rate. Moreover, the crystallization peak narrows for lower cooling rates, indicating that a shorter time is required for the polymer chains to crystallize.

#### 3.1.5. Effects of Shearing Time and Temperature on T_c_

**Figure 5:**
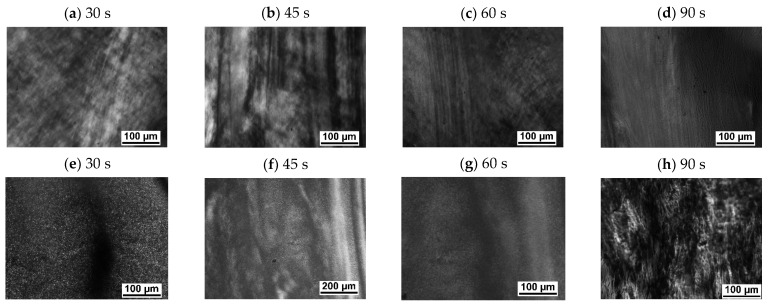
Effect of shearing time on chain orientation of (**a**–**d**) PBASGT and (**e**–**h**) PBAT samples, sheared before crystallization for 45 s at the cooling rate of 20 °C/min.

Figure 5 shows that, despite the non-sheared samples, which constitute an overall random orientation of polymer chains, the reorientation of chains takes place more effectively upon shearing and subsequently increasing the shear time as flow patterns appear for the sheared samples. It is found that applying shear at the rate 100 s^−1^ for 30, 45, 60 and 90 s to the PBAT samples increased T_c_ to 76 °C, 78 °C, 81 °C and 84.5 °C, respectively.

**Figure 6:**
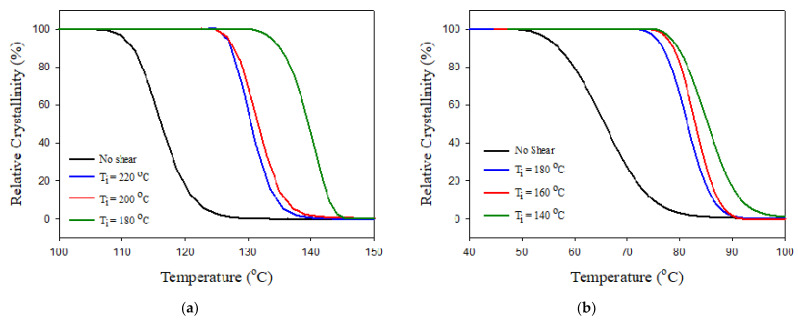
Effect of Ti on the crystallization of (**a**) PBASGT and (**b**) PBAT sheared for 45 s at 300 s^−1^ during cooling at 10 °C/min.

Figure 6 shows the impact of shearing temperature, Ti, on the crystallization of PBASGT sheared for 45 s at 300 s^−1^ during cooling at 10 °C/min. It turned out that, as the samples were sheared at lower temperatures, the induced crystallization happened more effectively than at higher temperatures. Applying the shear to the samples at 220 and 200 °C increased T_c_ by 4 and 10 °C, to the ranges of 130.4–131 °C and 135.6–136.5 °C, respectively. The significant increase was achieved by lowering Ti to 180 °C, where the highest value of T_c_, was obtained after shearing for 45 s. Shearing the specimen at 180 °C elevated T_c_ up to 140.4 °C during cooling at 10 °C/min. The shorter the time gap between the cessation of shearing and the onset of crystallization, the less possibility for crystal nuclei to re-melt at temperatures above Tm and for polymer chains to recoil and relax. For each shear rate, a critical temperature exists for initiating the shear. A further decrease in Ti leads to the solidification of the sample and its failure under shear.

### 3.2. Nanofiber-Nucleated Crystallization of PBAT

#### 3.2.1. Effect of CNFs on T_c_

**Figure 7:**
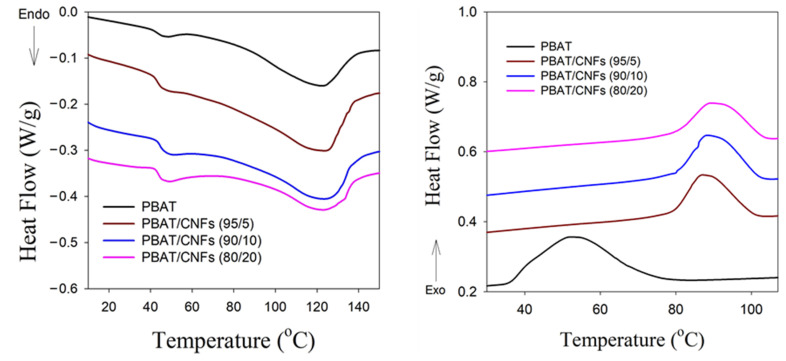
Melting endotherms and cooling exotherms of PBAT as well as PBAT/CNF blends with different concentrations of 5, 10 and 20 wt.% of CNFs.

Since PBAT is a commercial-grade material which is commonly used, knowledge of its crystallization temperature in the presence of bio-nanofibers is crucial for the in situ generation of biodegradable nanocomposites, which requires the crystallization of nanofibrous sheared inclusions at higher temperatures. For this purpose, the influence of cellulose nanofillers (CNFs) on the crystallization of PBAT was investigated based on the assumption that the introduction of CNFs may lead to an increase in the crystallization temperatures of PBAT. Figure 7 shows DSC curves for PBAT as well as PBAT/CNF blends with different concentrations of 5, 10 and 20 wt.% of CNFs after first heating and a subsequent cooling cycle without shearing. It was found that the addition of CNFs to PBAT had an insignificant effect on the degree of crystallinity (Figure 7a), whereas the presence of CNFs contributed to a noteworthy increase in the crystallization temperature of PBAT from 56 °C to 87–91 °C, depending on the content of CNFs. The greatest effect was achieved with 20 wt.% of CNFs, where the crystallization was shifted by 35 °C to 91 °C (Figure 7b).

#### 3.2.2. Effect of CNFs on thermal degradation

**Figure 8:**
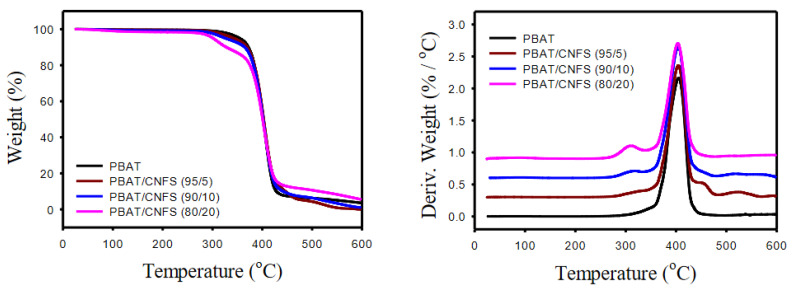
TGA and DTG curves of neat PBAT and PBAT/CNFs blends (with 5, 10 and 20 wt.% of CNFs), measured at the rate of 10 °C min^−1^.

We also attempted to evaluate the thermal stability of blends, where CNFs with concentrations of 5, 10 and 20 wt.% were incorporated in PBAT. Figure 8 reports the TGA and DTG results comparing the main peaks of neat PBAT and PBAT/CNF blends. The main peak observed for all the samples between 360 and 430 °C corresponds to the degradation temperature of PBAT, with a mass-loss rate between 93% for neat PBAT and 77% for PBAT/CNFs (80/20). The blends show this peak at 404 °C, which is almost the same as for neat PBAT at 405 °C. The subtle decrease in degradation temperature is attributed to the incorporation of the CNFs in the PBAT. Although PBAT/CNFs (95/5) are composed of two components, only one sharp and intense peak with an approximate loss of 93 wt.% was detected because the CNFs were well involved with the PBAT. Nevertheless, the thermal stability of the blend was maintained. Although the addition of CNFs with higher concentrations of 10 and 20 wt.% decreases the loss mass rate, it has no significant influence on the Tg, Tonset and Toffset values of this peak. Another peak, observed in the blends below 350 °C, corresponds to the degradation of CNFs at 315 °C and is intensified as the concentration of CNFs increases. The weight loss of PBAT/CNFS (95/5) due to the degradation of CNFs is insignificant however, the blends PBAT/CNFS (90/10) and (80/20) lose 5 and 10 wt.%, respectively.

In summary, when CNFs were linked to PBAT’s structure, the peak corresponding to the degradation of PBAT did not change. However, a new peak at 315 °C for blends were detected and intensified as the concentration of CNFs increased. In general, the PBAT/CNFs (95/5) presented very similar onset, peak and offset degradation temperatures (loss: ~ 93 wt.%) for the degradation of the PBAT phase. The degradation of the blends with 10 and 20 wt.% of CNFs occurred in a dual stage, where the samples were degraded in the first stage by 5 and 10 wt.%, respectively.

## 4. Conclusions

This study attempted to investigate the influence of the shearing conditions encountered during in situ generation of biodegradable nanocomposites on the non-isothermal crystallization of two copolyesters, PBASGT and PBAT. The induced nucleation upon shearing was accompanied by faster crystallization at elevated temperatures, whereas the growth of these nucleation sites, with higher density, was hindered, resulting in smaller lamella stacks. In addition, the nucleation was initiated at higher temperatures by cooling the melt slowly. This effect was more noteworthy at lower shear rates. Shearing the sample at lower temperatures, closer to the nominal melting point, was more effective. Despite the orientation of molten chains during shearing, the point-like nucleation was not altered. The effect of CNFs on the PBAT crystallization and degradation was elucidated. In comparison to PBAT, the PBAT/CNFs (95/5), with a shift in crystallization temperature by 35 °C, demonstrated similar onset, peak and offset degradation temperatures.

## Figures and Tables

**Table 1:** The effect of cooling rate on T_c_ of PBAT and PBASGT films sheared for 45 s.

Shear Rate (s^−1^)	T_c,PBAT_ (°C)		T_c,PBASGT_ (°C)	
	20 °C/min	10 °C/min	20 °C/min	10 °C/min
0	56.5	64.6	116.0	117.0
100	70.0	74.2	117.5	119.9
200	73.1	79.0	119.5	122.1
300	76.3	80.1	121.5	124.7
400	78.0	81.3	123.5	128.2
600	77.4	81.0	126.0	132.0
800	-	-	126.2	131.3

## Data Availability

Not applicable.
